# Retraction: Occult Non-small Cell Lung Cancer Presenting as Paraneoplastic Gastroparesis: A Case Report and Literature Review

**DOI:** 10.7759/cureus.r151

**Published:** 2024-11-07

**Authors:** Muhammad A Baig, Tejinder Randhawa, Muhammad B Majeed, Rohit Agrawal, Seema R Gandhi

**Affiliations:** 1 Gastroenterology, University of Illinois College of Medicine at Peoria, Peoria, USA; 2 Internal Medicine, John H. Stroger, Jr. Hospital of Cook County, Chicago, USA; 3 Internal Medicine, John H. Stroger Jr. Hospital of Cook County, Chicago, USA; 4 Gastroenterology and Hepatology, University of Illinois at Chicago, Chicago, USA; 5 Gastroenterology and Hepatology, John H. Stroger, Jr. Hospital of Cook County, Chicago, USA

This article has been retracted by the Editor-in-Chief due to image plagiarism. Figure 5 was reproduced from https://commons.wikimedia.org/wiki/File:Poorly_differentiated_non-small_cell_carcinoma_NOS_with_a_massive_host_inflammatory_response_-_Case_267_(8597445839).jpg Wikimedia Commons, but not properly cited and instead presented as a biopsy from the patient featured in the case report.

